# Supplementary material for: Identifying key features of digital resources used during online science practicals
Source: PLoS One. 2023 Oct 24;18(10):e0283054. doi: 10.1371/journal.pone.0283054 (PMC10597498; doi:10.1371/journal.pone.0283054)
Supplement: Attachment — Submitted filename: Response to Reviewers.docx [file pone.0283054.s002.docx]

Dear reviewers,

Thank you for the summary and the detailed comments from the reviewers.

We have edited the manuscript according to the comments, in particular we have unified the terminology, rewritten the introduction and added one analysis that allowed us to formulate a further conclusion. Detailed responses to each comment are provided below.

**Comments to the Author**

1. Is the manuscript technically sound, and do the data support the conclusions?

The manuscript must describe a technically sound piece of scientific research with data that supports the conclusions. Experiments must have been conducted rigorously, with appropriate controls, replication, and sample sizes. The conclusions must be drawn appropriately based on the data presented.

Reviewer #1: Partly

Reviewer #2: Partly

2. Has the statistical analysis been performed appropriately and rigorously?

Reviewer #1: No.

Reviewer #2: I Don't Know

3. Have the authors made all data underlying the findings in their manuscript fully available?

The [PLOS Data policy](http://www.plosone.org/static/policies.action#sharing) requires authors to make all data underlying the findings described in their manuscript fully available without restriction, with rare exception (please refer to the Data Availability Statement in the manuscript PDF file). The data should be provided as part of the manuscript or its supporting information, or deposited to a public repository. For example, in addition to summary statistics, the data points behind means, medians and variance measures should be available. If there are restrictions on publicly sharing data—e.g. participant privacy or use of data from a third party—those must be specified.

Reviewer #1: Yes

Reviewer #2: Yes

4. Is the manuscript presented in an intelligible fashion and written in standard English?

PLOS ONE does not copyedit accepted manuscripts, so the language in submitted articles must be clear, correct, and unambiguous. Any typographical or grammatical errors should be corrected at revision, so please note any specific errors here.

Reviewer #1: No

Reviewer #2: Yes

5. Review Comments to the Author

Please use the space provided to explain your answers to the questions above. You may also include additional comments for the author, including concerns about dual publication, research ethics, or publication ethics. (Please upload your review as an attachment if it exceeds 20,000 characters)

Reviewer #1: This article presents interesting research on the topic of science practical online teaching during the COVID-19 lockdown. The research was carried out as a part of the Erasmus+ project "My Home – My Science Lab". In the research, the data was collected from science teachers in Slovakia, Czechia, Slovenia, France, and Spain regarding shared web resources they used and would recommend.

While the study's topic is interesting, the article's language is unclear and difficult to understand.

1. It includes many errors, and I recommend that authors work with a writing coach or copyeditor to improve the flow and readability of the text. The paper should be written in an objective tone. Also, there are a lot of inconsistencies in the text, for example, the authors use both "eg." and "e.g." throughout the text, there is inconsistency in using small letters or caps (for example in Table 1), and inconsistency in the citation style used (for example line 507 with two different citation styles). Furthermore, for sentence separation the usage of ";" character instead comma is redundant, and the space character should be put after the comma, etc.

**Thank you for the comment, the whole text was checked and corrected, the citation style was unified.**

2. The authors are off to a good start, however, the study fails to address how the findings relate to previous research in this area. The authors should rewrite their Introduction to reference the related literature.

**The introduction was re-written to cover a wider context.**

3. The sample size is not very high. Also, it seems as if the data collection process should have been better organized since, for example, the authors don't know which web resources teachers have tried and possibly rejected.

**As we aimed at describing the digital resources which teachers used, we omitted the idea of asking for the resources they had tried and rejected. One can hardly assume that teachers, when searching for resources, will remember which resources they have not accepted, let alone attach relevant assessments to them retrospectively. . Based on the literature and also our results, we conclude (at least some) teachers were actively searching for new web resources during forced distant teaching (32 out of 89 web resources from our data set were discovered by the teachers during forced distant teaching). Still, there is a nearly endless supply of resources that is being broadened every day.**

4. Although the research results are interesting, there is a lack of serious contributions and conclusions drawn from the research, for example, the nature of how teachers incorporate the use of resources into their teaching and what kind of "digital element" they created from it.

**Thank you for your comment. These are interesting questions that mostly go beyond the scope we are working in. But based on it, we additionally analyzed the relationship between features of the digital resource and activity type (in which the resource was used by the teachers) and added it to the manuscript.**

Some more specific comments:
5. Page 5, line 89: I would recommend describing "recommendations for conducting online teaching" in more detail. **More detailed description added.**
Page 5, line 106: I would recommend describing "additional verbs typical for the digital environment" in more detail. **Examples added.**
Page 6, line 113: Missing reference supporting "have been shown to be a challenging method even in face-to-face settings." **Reference added.**
Page 10, line215: STEM is not a subject. **Corrected**.

6. Page 23, line 406: I suggest reorganizing the paper in order to provide a description of applications used for online teaching at the beginning. Also, giving more insight into existing applications, not only just the one mentioned by the surveyed teachers.

**A selection of the applications used was moved at the beginning of Results. We did not add a list or description of other existing applications as there is a huge number of them and we did not want to choose them subjectively based on our knowledge.**

7. Page 28, line 511: "i.e. the nature of how they incorporate its use into their teaching and what kind of ‘digital element’ they create from it, is enormous and we estimate that it exceeds the expectations of the creators of the resources" – this should be the base for the paper and would be interesting to see.

**We agree this would be interesting and we see it as a possible next step of research. However, to have the base for it, we wanted to describe the web resources in this manuscript. See comment 4**

8. Page 31, line 580: "We only collected the web resources the science teachers would recommend; consequently we don't know which web resources they have tried and possibly rejected. Therefore, we cannot distinguish which ones they don't want to use and which ones they don't know." – this should have also been addressed during the data collection.

**Added to “Sample and sampling”.**

Reviewer #2: The paper presents research on identifying characteristics of digital elements used in science practical to identify which digital elements characteristics can serve as markers for their acceptance by teachers.

9. **The authors should emphasize more the contribution of their research in relation to existing research, preferably at the end of the introduction.**

**Different characteristics are important for different perspectives that teachers use when preparing their lessons. See comment 4**

10. The Introduction and Theoretical background section provide background information for readers, including motivation for the research. However, this part lacks a clear definition of the term "digital element" and a description of the relationship between the terms "digital element", "resource", and "learning activity".

**Thank you, indeed. The terminology was clarified. The term online resource, digital resource, and resource is used in the meaning of a particular web page, and activity is one of the characteristics of the resources.**

**- see also comment 4**

11. The authors should distinguish the terms (concepts) "resource" and "learning activity". It seems that these terms are sometimes considered synonymous, which makes it difficult to follow the manuscript and understand the analysis performed and its results. In the introduction, the authors give the following examples of digital elements: videos, visual materials, and domain-specific simulations, but later they also mention investigations, experiments, games, modeling and construction, etc. as types of digital elements. For example, in the learning activity "knowledge revision", students can be referred to different web resources (e.g. a specific video), so it is necessary to pay attention to the terms used.

**Thank you, agree, see comments 4+10**

12. Also, before describing the importance of "science practicals", it would be desirable for the authors to describe in more detail what they mean by this term and provide some examples.

**The term science practicals has been defined and examples given.**

13.The description of the methodology does not specify the research questions. Formulating the research questions using appropriate terms would allow for better structuring of the sections in which the research findings and discussion are presented.

**Thank you, research questions were formulated and used for further structuring the text.**

14. Regarding the instrument, it is necessary to better justify the selection of the types of digital elements used to describe digital elements and to present all the statements used to classify them according to other criteria (e.g., pedagogical suitability).

**The selected characteristics of digital resources are described in theoretical background in more detail and the statements were added to methods - instrument.**

15. In the abstract, the sentence that starts with "We identified their key..." is unclear.

**The sentence was reformulated.**
