# Supplementary material for: Identifying key features of digital resources used during online science practicals
Source: PLoS One. 2023 Oct 24;18(10):e0283054. doi: 10.1371/journal.pone.0283054 (PMC10597498; doi:10.1371/journal.pone.0283054)
Supplement: Attachment — Submitted filename: Response to Reviewers.docx [file pone.0283054.s003.docx]

Dear editor and reviewers,

Thank you for the summary and the detailed comments.

We have reviewed our reference list, ensuring that it is accurate and up-to-date and contains no retracted references.

We revised the introduction and the entire manuscript to improve clarity. To ensure linguistic precision, we sought assistance from a native English speaker. Your feedback on specific cases was appreciated, and we addressed these concerns in our revised submission.

On behalf the author team,

Vanda Janštová
